# Implementation of direct health facility financing in the rural District of Kigoma in Western Tanzania

**DOI:** 10.11604/pamj.2023.46.19.41052

**Published:** 2023-09-14

**Authors:** Flora Joram, Jairos Hiliza, Sirili Nathanael, Amani Anaeli

**Affiliations:** 1 Department of Development Studies, School of Public Health and Social Sciences, Muhimbili University of Health and Allied Science, Dar es Salaam, Tanzania,; 2 World Health Organization, Kigoma Field Office, Kigoma, Tanzania

**Keywords:** Health financing, primary health care, health governance, Western Tanzania

## Abstract

The adoption of decentralization by devolution in Tanzania has enabled the implementation of a Direct Health Facility Financing (DHFF) program in the facilities. While copious gains have been reported under DHFF, there are also notable failures to improve health service provision. This study aims to explore the experience of implementing the DHFF program in the rural areas of the Kigoma District Council. An exploratory qualitative study was conducted in Primary Health Care (PHC) facilities of the Kigoma District Council. A purposive sampling technique was used to draw 21 key informants including leaders of health facilities and members of the Health Facility Governing Committees (HFGC). Key Informant Interviews (KII) were used to solicit information from the study participants. Content analysis technique was used to analyze data collected from study participants. Our findings present enablers and barriers in the implementation of DHFF. Successful implementation of DHFF was enabled by the availability of formal training and supportive supervision, adherence to DHFF guidelines, availability of planning guidelines at the health facility, functionality of the HFGC, and adherence to the procurement process. A low sense of ownership of the program, delays and insufficient fund disbursement, shortage of health workers, and inadequate knowledge of DHFF program implementation emerged as the barriers that impeded successful program implementation. Evaluating the implementation experience of the DHFF program requires policymakers at the national level to devise a mechanism for the timely disbursement of funds, reinforcing capacity building to increase the autonomy of health facilities in their daily operations. Furthermore, structural and operational barriers warrant further operational and implementation research.

## Introduction

Health financing is an important component of health systems aiming to improve health by delivering quality services. However, this may be hampered if global and local resources are not efficiently managed [1]. Literature suggests that in low-and-middle-income countries, low allocation of funds has led to impediment and stagnation of improvement and provision of quality service [2,3].

To achieve the “Health for All by the Year 2000” strategy, World Health Organization (WHO) members adopted Primary Health Care (PHC) as the main core policy as declared in the Alma-Ata in 1978 [4]. Despite the substantial increase of funds from external and internal sources, the available fee is still insufficient to improve health services [5]. To improve the health system performance, Tanzania like any other third-world country has experienced reforms that included health financing reforms. The health financing reforms entailed among other things, the introduction of cost-sharing in 1993, and the introduction of Community Health Funds (CHF) in 1996 [6].

The introduction of decentralization by devolution (D by D) dated in the 1980s led to the adoption of three levels of primary, secondary, and tertiary health care within the health system as part of the reforms [7,8]. Emphasis was made on the primary health level as the main point of entry for service provision and implementation of programs [9]. The devolution led to the introduction of the DHFF program to improve the allocative efficiency of funds and expenditure to be shaped by user preference [10]. The D by D enables the government to grant autonomy to healthcare facilities in the planning, fund management, procurement, and expenditure reporting of financial use and management [11].

Direct Health Facility Financing (DHFF) is defined as the direct provision of government or external funds to the health facility to meet operational requirements. The facility prepares a budget and once approved, is spent by the facilities [11,12]. Direct Health Facility Financing (DHFF) reform program aims to improve health system performance through disbursing funds to the primary health care facilities to improve the delivery of health services and achieve universal health coverage (UHC) [13]. However, its achievement has been hindered by understaffing, inadequate training, delay in funds disbursement, and health facilities' geographical location context [5]. This has led to the adaptation of several financing approaches to meet PHC objectives including Result-Based Financing (RBF), facility funding by revolving funds, and DHFF.

In Tanzania, over two-thirds of the population reside in rural areas and rely on primary health facilities for service provision with only 55% of nurses, and 25% of physicians working in rural areas [14,15]. After the adoption of DHFF in 2016, implementation of the DHFF program began fully in 2018 in Tanzania in all districts, under the guidance of the President's Office-Regional Administration and Local Government (PO-RALG). The DHFF depends on funds disbursed from the health basket funds, National Health Insurance Fund (NHIF), improved community health fund, and cost-sharing user fees to support health facilities' priorities as well as ensure resource allocation upon program areas [6].

Despite the implementation of DHFF by the Tanzania government, low improvements in the primary health facilities have been observed, and an overburden of workload. The shortage of the health workforce is seen to be about 50% with a mean attrition rate of 15.3% and no reporting rate of 26% [16]. This situation is worse in rural areas where the available workforce performs multiple duties including patient care, data management, and managerial tasks. Despite the task, most healthcare providers have low financial management and procurement skills.

Therefore, despite the reforms and different strategies adopted for DHFF, primary health facilities have failed to improve service provision, thus challenging the realization of the health sector reforms goals [17]. As such The DHFF implementation program is designed to improve the quality of health services, improve efficiency, and mobilize health workers and community levels for improved health services. However, impediments of the achievement of the program goals in primary healthcare facilities still exist. The study aimed to explore the experiences in the implementation of the DHFF program at primary healthcare facilities in the Kigoma district council.

## Methods

**Study design:** this study was an exploratory qualitative study design [18] conducted in the Kigoma district council from August 2019 to April 2020. The exploratory qualitative study design was considered due to the nature of the study which needed an in-depth understanding of the program and aimed at exploring the experience of implementing the DHFF program.

**Study context:** Tanzania coordinates and implements all health services under the Ministry of Health (MoH) while actual implementation of service delivery is done by the President´s Office-Regional Administration and Local Government (PO-RALG). Concerning DHFF, PO-RALG has a decentralized structure for the management of health services with the Regional Health Management Team (RHMT) at the regional level being responsible for conducting supportive supervision and mentorship for the district councils on issues related to the DHFF program implementation.

At the council level, CHMT is responsible for ensuring that the DHFF program is implemented, providing technical assistance to primary healthcare facilities including financial management and preparation of annual plans as well as budgeting for the individual health facilities [6]. The Facility Management Teams and Health Facility Governance Committees (HFGCs) have responsibility for planning, procurement, and budgeting for the health facilities in addition to the endorsement of all transactions at these primary health facilities.

This study was conducted in the Kigoma district council which is located on the shores of Lake Tanganyika and is among the eight councils in the Kigoma region. Kigoma district council was purposefully selected among eight councils in Kigoma due to its complex geographical characteristics between health facilities as compared to other councils. According to the Tanzania Health Management Information System (HMIS), Kigoma district council has a total of 46 health facilities of which 34 (74%) are publicly owned; two health centers and 32 dispensaries, which implement a direct health facility funding program. Notably, some health facilities are hard to reach due to transport challenges, with poor retention of health workers, and a shortage of social services such as clean water, education, internet, and better health services.

**Study participants:** study participants involved those who were directly involved in the implementation of the DHFF program at primary health facilities. This included health facility in-charges, nurse in-charge, and members of the Health Facility Governing Committee (HFGC).

**Sampling strategies and sample size:** a purposive sampling technique was used to identify seven primary health facilities and rich-information participants. The seven health facilities were selected from other health facilities depending on their geographical location and they were assigned letters A, B, C, D, E, F, G, and from each health facility, three key informants were interviewed based on their positions and responsibilities concerning the implementation of the DHFF program at the primary health facilities. Whereas facilities A and B were health centers, the rest of them were dispensaries. Regarding the facilities located far away from the district council, a total of three health facilities were selected in hard-to-reach areas along Lake Tanganyika including facilities A, C, and G. For facilities located in accessible areas to the district center, a total of four facilities were selected including facilities B, D, E, and F. For each selected health facility, a minimum of 3 selected people was invited for KII.

**Data collection:** using a Kiswahili semi-structured interview guide was piloted at Ujiji Health Center and Buhanda Dispensary in Kigoma municipality to the respective targeted participants. After the Amendments, the final tool was adopted. We conducted 21 Key Informants´ Interviews (KIIs) from seven selected facilities (Table 1). The team of researchers conducted all interviews and moderation while the research assistants took notes and audio recordings of the interview. All KIIs were recorded using a tape recorder. The tape recorder was locked in a secured place by the researcher. On average, the KIIs lasted about 50 minutes and interviews. Interview schedules were sought before the interview day. The interview was carried out in the natural settings of informants including their offices at the facilities.

**Table 1:** distribution of key informants by their titles

Category KIIs	Male	Female	Total
Health facility in-charge	4	2	6
Matron/patron	2	3	5
HFGC chairperson	5	0	5
HFGC members	2	3	5
Total	13	8	21

KII: Key Informant Interviews; HFGC: Health Facility Governing Committees

**Data analysis:** all KIIs were transcribed verbatim and translated into the English language. To maintain the original meaning, the first author conducted a back-translation of the transcripts. Codes, sub-categories, and categories were generated. In cross-checking interpretation and reliability, an independent researcher who was not part of the study checked the coding to cross-check interpretation and reliability. Content analysis was used to analyze data through an inductive approach. A codebook was developed based on the study objective. Updating of the codebook was conducted iteratively as categories emerged. The codebook was discussed by other authors and the final agreed codebook was imported into the NVivo software version 11 qualitative data analysis software.

Through identifying similarities and differences between codes, sorting was conducted to derive subcategories that were further aligned to form categories. The analysis process was conducted repeatedly to identify patterns that derived from a specific category. The last process included the presentation of categories with brief verbatim quotes that define the meaning underpinning each category.

**Ethics approval and consent to participate:** ethical clearance for this study was obtained from the Muhimbili University of Health and Allied Sciences Institutional Review Board (reference number DA.287/298/01A), as it is stated in the ethics approval letter as “the chairman has, on behalf of the senate approved ethical clearance for the above-mentioned study”. The permission for data collection was obtained from Kigoma regional administrative secretary, Kigoma district council executive director, the district medical officer, and the facilities in charge. Written informed consent was obtained from the participants before KII was conducted. To assure confidentiality, the interviews were conducted in a comfortable place identified by the participants themselves. All participants were assigned unique identifiers for the concealment of identity. Generally, the study was conducted per regulations and guidelines stipulated in the Helsinki Declaration as it was revised in 2008 by granting the study participants the autonomy to participate or withdraw from the study at any time during the study period.

## Results

We present findings emanating from interviews with 21 key informants distributed in 7 health facilities. The age of the participants ranges from 23 to 64 years across all study participants groups as follows: health facility in-charge from 27 to 48 years, matron/patron from 23 to 54 years, HFGC 35 to 64 years. Three categories emerged from the analysis of the experiences of implementers on the implementation of the DHFF program in primary health facilities of the Kigoma district council. The categories included the existence of formal training and supportive supervision, adherence to DHFF guidelines, and the existence of barriers that hinder the smooth implementation of the DHFF program (Table 2).

**Table 2:** summary of findings showing subcategory and category

Subcategories	Category
1. The usefulness of formal training on DHFF implementation	Existence of formal training and supportive supervision towards the implementation of the DHFF program
2. Supportive supervision from CHMTs is crucial for DHFF implementation	
1. Availability of planning guidelines at the health facility	Adherence to DHFF guidelines
2. The functionality of HFGC	
3. Adherence to the Procurement process	
1. Lack of sense of ownership of the program	The existence of barriers that hinder the smooth implementation of the DHFF program
2. Delays in fund disbursement	
3. Inadequate health workers	
4. Insufficient for fund disbursement	
5. Low knowledge of program implementation	

DHFF: direct health facility financing; CHMTs: council health management teams; HFGC: health facility governing committees;

**Existence of formal training and supportive supervision towards the implementation of the DHFF program:** the existence of formal training and supervision among implementers refers to all activities on training and supervision of the DHFF program that have been stipulated in the DHFF guidelines. Such activities have been viewed by participants as essential to instill competence in the implementation of the program. The attributes revealed included the usefulness of formal training on DHFF implementation and supportive supervision from CHMTs which is crucial for DHFF implementation.

**The usefulness of formal training on DHFF implementation:** formal training was reported by the participants as being crucial in the implementation of the DHFF program as it contributes to the competence in financial and procurement operations at the facility. Participants who received formal training using DHFF guidelines were more confident in executing financial and procurement tasks as stipulated in the guidelines. *“Honestly, without training, I could not be able to do anything with this program. The training helped much as you can see, I have no background in financial management, but now because of training I can handle and manage the funds we get following the directions”* (health facility in charge for facility C).

More so, participants who did not receive formal training reported having difficulties in performing DHFF activities hence affecting the health facilities' performance. However, most participants reported having several on-site mentorships through supportive supervision which are conducted by the CHMT on regular basis. Notably, onsite mentorship was reported to play a key role in implementing DHFF. *“Aaaaa…! no I did not receive any formal training before the DHFF program, but we receive on-site mentorship when they (CHMT) come for supervision, and even when we send documents to DMO for signing they correct us where we have done wrongly. In this way, we learn! We also get support from those who receive training as we ask them if we face any difficulty”* (matron health facility D).

**Supportive supervision from CHMT is crucial for DHFF implementation:** participants reported supportive supervision was pivotal in supporting DHFF program implementation at the primary health facilities. Implementation of support supervision was back and forth from the CHMT to the health facilities using scheduled and non-scheduled visits on regular basis. Further, participants reported visiting the CHMT office, especially during planning for procurement, and other DHFF fund expenditures. *“At the DMO office, they have a schedule to visit our facility for supportive supervision normally and it is quarterly basis. Further, the accountant and DHFF coordinator come to our facility regularly, especially after the DHFF funds have been deposited into our facility account”* (health facility in charge of health facility D).

Support supervision has had an impact on the performance of the healthcare workers in the facilities. Our findings show that this process has strengthened the stringent adherence to guidelines as well as imparted new updates to the healthcare workers and mentorship on how to write proper financial reports. *“To prepare program report is difficult, but they (CHMT) come to our facility for supportive supervision, they monitor and coach us on how to prepare the DHFF program implementation report”* (matron for facility E). However, the HFGC members were found not to be involved during the support supervision and hence not much training and mentorship is imparted to them. *“Frankly speaking, I have not been supervised by the CHMT, but I only see government staff from the district come here regularly”* (HFGC member of facility A).

**Adherence to DHFF guidelines:** managers of the health facilities and HFGC reported that adherence to the stipulated guidelines is a key driver for the successful implementation of the DHFF program. This process ensures collective accountability and sustainability of the program. The outspoken attributes for adherence to DHFF guidelines were regular referencing of the available DHFF guidelines during the planning and implementation of the DHFF activities. Further, the involvement of the HFGC in all activities as stipulated in the guidelines was among the frequently discussed topics.

**Availability of planning guidelines at the health facility:** our result shows that having DHFF guidelines at the facilities helped the implementers in priority setting and involvement of the HFGC in the preparation of comprehensive council health plans (CCHP). The participants felt that the availability of the guidelines supported the positive implementation of the DHFF program. *“Of course, yes, we use a guide to prepare a plan, which after being approved by PO-RALG through DMO they return to us, therefore when we want to implement the program, every quarter. I use the approved plan, and guidelines from PO-LARG, for example, for the April-June quarter you have to implement certain activities depending on the initial plan, therefore, I --use them to guide and plan approved by PO-RALG...”* (health facility in charge for facility E).

**The functionality of health facility governing committees:** our results show that the health facility in charge and HFGC members reported the involvement of the HFGC in the planning and implementation of all activities related to finance and procurement as directed by the DHFF guidelines. Further, it was reported that the HFGC are signatories in approving and supervising daily DHFF facility plans and procurement processes. *“The letter from the DMO comes to the facility in charge intending to convene a meeting aimed to prepare a procurement plan. They cannot implement anything without involving us because I have a place to sign, and truly I can say that I am aware of everything which is done at the facility concerning DHFF”* (HFGC chairperson for facility A).

**Adherence to the procurement process:** the health facility in charge and HFGC members reported adherence to the DHFF guidelines in the procurement process including planning, bidding, and reception of the delivered supplies. Furthermore, results show that all these activities involve the HFGC in which they have meetings to discuss what and where to procure. Competitive bidding is employed to allow transparency and obtain the best market value for the supply and the order is executed using the Local Purchasing Order (LPO) as directed by the DHFF guidelines. *“...... If we want to buy stationeries or anything, the committee sits after the funds have been deposited in our facility bank account and decide, for example, one hundred thousand is for buying gas as it is in the facility plan, after agreement from committee members we go to the market to conduct bidding, we use the guide of 3 bidders, based on the market prices, then we choose the one who has a low price compared to others”* (health facility in charge for facility C).

**Existence of barriers that hinder the smooth implementation of the DHFF program:** barriers that are deemed to impede the facility operations, as well as sustainability in the implementation of DHFF, emerged in this study. The barriers were due to the incompetence of the healthcare workers and the shortcomings of the health system in implementing the DHFF program. The barriers included a low sense of ownership among program implementers, delays and insufficient funds disbursed, inadequate health workers, and inadequate knowledge of program implementation.

**Low sense of ownership of the program:** a low sense of ownership of the program is among the pronounced barriers. Despite the direct deposit of funds to the facility bank account, the use of the allocated funds needs approval from the District Medical Officer (DMO). Furthermore, the activities that are planned at the facilities, need approval from the Council Health Management Team (CHMT). The experience from the health facility implementers has shown that some activities in the hospital are denied by the DMO or do not receive approval from the CHMT. *“There are many challenges, first funds are deposited into our accounts (facility accounts) but in a real sense, they (CHMTs) are still the owners of the funds. They are the ones who approve of how to use the funds. It reaches a stage when we see that the facility needs a certain thing we want to buy, but they say no you cannot buy even if it is in the plan, this is a challenge”* (health facility in charge of facility B).

**Delays and insufficient fund disbursement:** delay in the disbursement of funds is one of the barriers affecting DHFF program performance as a disbursement is done after the ending of other quarters. Delay in funds lenders delays in activities implementation and pilling up of activities in one quarter. This affects the quality of services at the health facility. Health facility in charge for facility E: *“…it can happen that all the funds deposited at the same time, for example, funds that cover three quarters deposited into the facility bank account at once. Therefore, we have to implement the activities of all three quarters. This affects much of our normal work performance at the facility as we have to spare extra time to finish those activities. And most of the time, we were out of stock for essential supplies”*.

Insufficient funds disbursed have been reported as a barrier, especially in the covering of daily expenditures for the facilities. Participants reported the funds distributed to the facility do not always align and fall short of the planned amount in the Council Health Operation Plan (CHOP). Health facility in charge for facility D: *“…insufficient amount of funds is deposited into the account. The fund is not enough considering the needs of the facility for example here at my facility there is a high population of people who seek service here”*.

**Shortage of health workers:** a shortage of healthcare workers has been reported by participants as hindering the effective implementation of the DHFF program. This has been noted as affecting accessibility to service to clients/patients visiting the facilities as the provider has to divide time between administrative and clinical service duties. *“Honestly speaking; this program affects my responsibility at the facility. I also use nighttime to compensate for the time so that I at least get time for the patients at the facility”* (health facility in charge of facility B).

The double burden on the facilities is also added due to the understaffing at the district office, which revealed that only two accountants support 32 health facilities in mentorship and assessment of financial plans and implementation. This leads to delays and interference with the facility work schedule due to follow-ups. Health facility in charge for facility F: *“…you can have a document that has already been signed by the District Executive Officer (DED) when it comes a time to go to the accountant, you find that she has many files that she is supposed to work with, and there are more than 30 health facilities that they work with”*.

**Inadequate knowledge of DHFF program implementation:** knowledge regarding the DHFF program was noted to be lacking among implementers. Participants reported incompetence at the district and facility levels and across the program implementation chain. The most challenging issue is the interpretation of the regulations on DHFF funds by both facility managers and CHMT supervisors and mentors. This impediment in the flow of knowledge and skills was reported by participants to have reduced the effective implementation of the DHFF program. Health facility in charge of facility B: *“…even those who supervise us have no direct technical knowledge concerning the program. If you go with the document today, they can show you how to do it and if you go back tomorrow to the same person, he/she changes the rules. This reflects their little knowledge of the rules and regulations of the program......”*.

Moreover, incompetence and unclear stipulation of work delegated in accounting skills by the facility managers is yet another barrier as it was reported to cause hesitancy in the implementation of activities due to long consultations or repeated directives. Patron at facility F: *“…most of the health facility in-charge perform tasks that were not employed for. And they were not taught to be accountants or procurement officers at the health facility”*.

## Discussion

This study set out with the aim to explore the experiences of the implementation of the DHFF program at primary health facilities in the Kigoma district council. It is interesting to note that the existence of enabling factors led to the successful implementation of the DHFF program within the district. It further depends on how barriers are handled. The study findings are interpreted in line with the DHFF implementation program. According to DHFF conceptual framework, when implementers receive training and supportive supervision, adhere to program guidelines, and manage barriers to program implementation leads to the successful implementation of the DHFF program.

**Enablers experienced during the implementation of the DHFF program:** the study findings reveal training and supportive supervision enable the implementation of the DHFF program in achieving targeted goals. Training and supportive supervision are essential in adding knowledge, and information to implement the program. While the study reveals implementers valued formal training as it was helpful in DHFF operation. Supportive supervision was provided as per scheduled and non-scheduled enabled implementers in work plan approvals for program implementation. A study argued that when implementers receive training and supportive supervision, adhering to program guidelines leads to the successful implementation of the DHFF program [6,19].

A study in Kenya [11] revealed training leads to sustainable implementation of direct facility-based financing, pointing out that regular capacity building in terms of skills knowledge development, and programmatic guidelines leads to successful program implementation and sustainability. These findings are in line with studies conducted in China and Kenya [20,21]. Our study reveals that the availability of active HFGC members resulted in adherence to program guidelines and the procurement process enhanced the accountability and sustainability of health workers. Further, findings show a reduction of bureaucracy in the implementation of health plans, as most of the decisions about health plans are locally made without necessarily involving district leadership. This has led to the empowerment of implementers at the PHC levels to be independent in decision-making. Similar findings were reported in studies conducted in China, Tanzania, and Kenya [11,20,22,23].

**Barriers experienced during the implementation of the DHFF program:** in reviewing the literature, the findings of these studies support evidence from previous studies that have shown barriers experienced during the implementation of the DHFF program in Tanzania and other contexts. Findings from our study show requirements for approval of funds and activities are planned by the district leadership, which may result in denying or late approval of funds. These results in hampering the program. Similar findings were reported from Tanzania [24], indicating that low ownership among implementers of the DHFF program affects the quality of health services since there is no full autonomy.

In this study, the disbursement of funds plays a critical role in the implementation of DHFF programs. Delay in the disbursement of funds is the limitation in autonomy portrayed within the PHC facilities in the implementation of DHFF programs. The provision of quality health services reflects the availability of funds promptly within the PHC level. The observable cause of delays is the dependence on donor funds and the existence of government priorities. These similar findings were reported in the Kongwa District in Tanzania, where delays in disbursement caused difficulties in the implementation of health-related activities [19]. Further, like our findings, the study emphasized the provision of full autonomy to the PHC level to implement DHFF programs. This was also observed in Kenya [21], in a study that reported that PHC facilities do not have managerial and financial autonomy which negatively impacts service delivery quality and health facility efficiency.

The most obvious finding to emerge from the analysis is that understaffing has led to the low accessibility of services by clients/patients visiting the facilities due to an increase in the workload of the health workers. The shortage of health workers is an inappropriate staffing level in the primary health care facilities affecting the DHFF implementation program. A study conducted in Tanzania [19] revealed insufficient and unqualified health workers impeded successful program implementation. Another study in the Kaliua District of Tanzania [22] showed staff shortage hindered the targeted objective of improving the quality of service delivery as intended within the program.

The study demonstrates clearly how inadequate knowledge hinders the effective achievement of DHFF program implementation. Inadequate knowledge is a lack of qualities and abilities to implement the DHFF program. While the low interpretation of the regulations on DHFF funds and unclear stipulation of work delegated results in low management of funds, implementers acknowledge low knowledge among the district leadership. This concurs with a study conducted in the Kongwa District in Tanzania [19], which showed limited knowledge and skills in planning among district leaders resulted in the unsuccessful implementation of DHFF in the PHC facilities. Further, a study conducted in Tanzania [13], on decentralization and the healthcare prioritization process revealed that district leaders dominate priority settings for PHC facilities, resulting in hindering the implementation process.

**Study limitation:** the selection of one district limited the generalizability of the findings. However, the selection of the facilities was done purposefully to cover both hard-to-reach and accessible facilities which represents the actual situation in most districts in Tanzania [6]. Another limitation was due to the use of only key informant interviews for the collection of data which is subject to interviewer and information bias. To alleviate this, we had a key informant diversity that included the healthcare managers and HFGC members. Without contradicting the above narration, this study finding provides insight into the situation in most districts in Tanzania.

## Conclusion

The findings of this study have provided insight into the facilitators and barriers to the DHFF implementation program. Health system strengthening at the primary health facilities can be a worthwhile investment in improving DHFF program implementation. This first and foremost requires financial managers at the national level to devise a mechanism for timely fund disbursement, and program implementers at the national level to reinforce capacity building to the regional and council health management teams as well as devise a mechanism to increase the autonomy of the health facilities to execute the DHFF program. These efforts should be in synergy with the CHMT´s consistent adherence to the supervision schedule for conducting mentorship and performance evaluations of the facilities. Moreover, a collaborative effort among all stakeholders is key to achieving desired outcomes but this must be reinforced with outstanding stakeholder engagement strategies that consider contextual considerations of the specific facilities. Furthermore, structural and operational barriers warrant further operational and implementation research.

### What is known about this topic

- *The significance of direct health facility finance the to increase accountability at the facility level on financial management;*
- *Direct Health Facility Financing system reduces the bureaucratic processes and procedures for finance expenditure*.

### What this study adds

- *The healthcare providers at health facilities, are not meant to manage funds, so this study has shown;*
- *The need to build the capacity of healthcare providers at the facility level on financial and procurement management;*
- *Delay in fund disbursement in the health facility bank account, negatively impact DHFF intended results; the health facility incharges, lack the autonomy on the DHFF program management; and inadequate human resources for health, compromises the implementation of the DHFF program*.
